# Marijuana: Current Concepts^†^

**DOI:** 10.3389/fpubh.2013.00042

**Published:** 2013-10-10

**Authors:** Donald E. Greydanus, Elizabeth K. Hawver, Megan M. Greydanus, Joav Merrick

**Affiliations:** ^1^Department of Pediatric and Adolescent Medicine, Western Michigan University School of Medicine, Kalamazoo, MI, USA; ^2^National Institute of Child Health and Human Development, Jerusalem, Israel; ^3^Health Services, Division for Intellectual and Developmental Disabilities, Ministry of Social Affairs and Social Services, Jerusalem, Israel; ^4^Division of Pediatrics, Hadassah Hebrew University Medical Center, Mt. Scopus Campus, Jerusalem, Israel; ^5^Kentucky Children’s Hospital, University of Kentucky College of Medicine, Lexington, KY, USA

**Keywords:** public health, cannabis, marijuana, abuse, dependence, withdrawal

## Abstract

Marijuana (cannabis) remains a controversial drug in the twenty-first century. This paper considers current research on use of *Cannabis sativa* and its constituents such as the cannabinoids. Topics reviewed include prevalence of cannabis (pot) use, other drugs consumed with pot, the endocannabinoid system, use of medicinal marijuana, medical adverse effects of cannabis, and psychiatric adverse effects of cannabis use. Treatment of cannabis withdrawal and dependence is difficult and remains mainly based on psychological therapy; current research on pharmacologic management of problems related to cannabis consumption is also considered. The potential role of specific cannabinoids for medical benefit will be revealed as the twenty-first century matures. However, potential dangerous adverse effects from smoking marijuana are well known and should be clearly taught to a public that is often confused by a media-driven, though false message and promise of benign pot consumption.

## Introduction

A number of chemicals are inhaled for the development of euphoria, including marijuana (cannabis, pot), methamphetamine, heroin, crack cocaine, phencyclidine, and nitrites (amyl and butyl) (1). Marijuana (cannabis, pot) has been known to *Homo sapiens* for thousands of years and concern has been raised over the past two centuries regarding its potential adverse effects – leading to various laws in the United States and other countries to control its production and use (2) (see the Preface). The psychoactive chemical, *delta-9-tetrahydrocannabinol* (THC) was isolated in the mid-1960s while other aspects of the endocannabinoid system [cannabinoid receptors (CB1 and CB2) and key endogenous cannabinoids (2-arachidonoyl glycerol and anandamide)] were identified over 20 years later. Though use of phytocannabinoids are being increasing linked to improvement of some health conditions, frequent users of cannabis are at increased risk for adverse effects which can lead to additional health problems (3). Research is identifying components of cannabis which are not psychoactive and may become established parts of the pharmacopeia as the twenty-first century continues.

## *Cannabis sativa* Plant

The products of cannabis are made from the easily grown hemp plant, *Cannabis sativa*, and the psychoactive ingredient, *delta-9-THC*, is at the heart of the complex cannabis controversy in this and the past century. The euphoria can last minutes to hours. The enzyme, Δ1-tetrahydrocannabinolic acid synthase, catalyzes the oxidative cyclization of cannabigerolic acid (CBGA) into Δ1-tetrahydrocannabinolic acid, which is the precursor of THC (4). This enzyme controls the psychoactivity of *C. sativa* (4) due to THC, which is present in the *C. sativa’s* dried leaves, seeds, stems, flowers (sensimilla), and oil (5). The pot of the 1960s–1970s contained 1–2% THC, while the Hawaiian sensimilla product was 3%; this is in contrast to current versions with much higher THC percentage as measured by the Potency Monitoring Project and other research (5, 6). A recent survey in Japan revealed an average potency of 11.2% with a maximum potency of 22.6% (7). High-potency cannabis is referred to as “skunk.”

Cannabis remains a popular global drug that is easily obtained around the world. Movements are increasing to legalize this drug, because of the research noting positive medical benefits as well as the intense euphoria it produces along with a popular, though false impression that this is a “safe” drug (8, 9). Cannabis remains a controversial drug, because its illegal status places it in conflict with the popular notion that pot is a harmless chemical – a notion made fashionable or trendy by many media and Hollywood personalities who may promote its use to enhance their *joie de vivre*. Such promotions seem to be effective as noted by the popularity of this illicit drug. However, marijuana (cannabis) has been prohibited in the United States since the 1937 Marijuana Tax Act as a federal law and is classified by the US Drug Enforcement Agency (DEA) as an illegal Schedule I drug. Some states in the US have legalized small amounts of cannabis that a person can carry without forensic consequences.

## Prevalence

Prevalence data for cannabis can be determined in various ways such as self-report data, waste water (sewage) analysis, and sales of cigarette paper (10, 11). Research notes that marijuana is the most commonly used illicit drug on earth (12, 13). The most frequently used substances among American adolescents are tobacco, alcohol, and marijuana. Marijuana (“*weed, pot, hash, BC Bud, Ganja, grass, smoke, doobs*,” others) is an illicit schedule I drug that constitutes about three-fourths of the illegal drug utilization in the United States (14–16).

Many studies have been done to confirm the high use of cannabis among adolescents and young adults of the world. The 2007 European School Survey Project on Alcohol and other Drugs (ESPAD) reported that life-time use of cannabis among students (age 15–16 years) in Europe ranged from 3% in Armenia to 45% in the Czech Republic with an average of 19% among 35 countries (see Table 1). One-third of Canadian university students used cannabis (17).

**Table 1:** **Life-time use of marijuana: 2007 ESPAD (18) (15–16 year olds)**.

	%
Armenia	3
Austria	17
Belgium (Flanders)	24
Bulgaria	22
Croatia	18
Cyrus	5
Czech Republic	45
Estonia	26
Faroe Islands	6
Finland	8
France	31
Germany	20
Greece	6
Hungary	13
Iceland	9
Ireland	20
Isle of Man	34
Italy	23
Latvia	18
Lithuania	18
Malta	13
Monaco	28
Netherlands	28
Norway	6
Poland	16
Portugal	13
Romania	4
Russia	19
Slovak Republic	32
Slovenia	22
Sweden	7
Switzerland	33
Ukraine	14
United Kingdom	29
Average	19

The United States Centers for Disease and Prevention (CDC) YRBS (Youth Risk Behavioral Surveillance) reported a life-time use (once or more times) among US high school students of 31.3% in 1991 that increased to 47.2% in 1999 and was 36.8% in 2009 (19). Use of marijuana by US high schools students that occurred 30 days before the survey ranged from 14.7% in 1991 to 26.7% in 1999 and 20.8% in 2009. Other studies note marijuana use by adolescents that range from 28% in New York City versus 38% across the United States (20). Prevalence of cannabis use disorders have increased in veterans in the United States and were higher in states which allowed cannabis for medical purposes (21).

### Consumption of cannabis (marijuana)

Marijuana is typically smoked as a *joint*, but can be taken orally in various foods, teas, or capsules which may be used as “medicinal” marijuana. It can be prepared in food for oral consumptions, as in brownies, cookies, or spaghetti. Various oral consumptions are found in different countries. For example, in eastern Iran, there is a special solid “pie” called *Majoon Birjandi*, which is consumed by adolescents to reach a cannabis-induced euphoria (22).

The marijuana (bhang) cigarette is rolled from the *C sativa* plant (upper leaves, tops, and stems) that is cut and dried. *Hashish* refers to dried exudate that comes from the top and underside of the plant leaves while concentrated hashish distillate is called *hashish oil*. *Sensimilla* is another potent marihuana product that is made from the seedless female flower of the cannabis plant. The “typical” marijuana cigarette includes about 20 mg of THC that is produced from a gram of *C sativa* leaves and buds; however, much variation in potency can be found (*vida supra*). THC can be found in the body for up to 2 weeks after use of a single pot cigarette. A *blunt* is a cigarette or cigar form made from tobacco and filled with marijuana in a process called *boosting*; this can be used to boost the effects of other drugs such as alcohol (23).

### Cannabis and other drug use

Pot is often combined with consumption of alcohol or diazepam which increases cannabis sedative effects. Addition of various other drugs enhances the euphoric effects. For example, marijuana is often mixed with various drugs such as nicotine, cocaine, opioids, or hallucinogens [as lysergic acid diethylamide (LSD)]. It is also mixed with drugs such as phencyclidine (PCP) in which the joint is dipped (hand-rolled) into PCP dissolved in an organic solvent (as formaldehyde); after drying it is smoked – called “*water, wet, or Sherms*.” Such additions add to the complications of pot use. Other additives include glutethimide and methaqualone, which have been popularized in the past. Persons abusing marijuana who also take disulfiram due to alcohol abuse can develop increased psychoactive effects of cannabis due to THC blockage by the disulfiram (24).

Cannabis users also smoke tobacco and research claimed that this occurs for a variety of reasons, including shared genetic factors, the similar cue of smoking for both, and withdrawal symptoms that are seen to some extent in both (25). One study of 467 adults who regularly smoked both cannabis and tobacco found that one-third started with cannabis-first, nearly half initiated tobacco before cannabis, and most cannabis smokers who stopped tobacco did so after taking up regular cannabis consumption (26).

As noted, those who consume marijuana also tend to use other drugs and it may serve as a gateway drug from early experimentation in adolescence to other drug use (27, 28). For example, two different studies reported that 45% of college students who illegally used prescription drugs also abused marijuana, while 24–57% also abused alcohol (29). Cannabis initiation usually follows alcohol use though cannabis use can start before alcohol use and African-Americans (versus European-Americans) have an increased risk for this cannabis-first trend (30). Other research notes increased risk for cannabis-related problems in African-American females versus European-Americans (30). Some research identifies declining socioeconomic position from childhood to adulthood as a risk factor for cannabis as well as tobacco consumption (31).

Some researchers reported that patients seen in a pain clinic were at increased risk for use of marijuana; for example, one study of these patients with 21, 746 urine specimens found 13.0% incidence of urine with cannabis (THC); also, 4.6% were positive for cocaine and 1.07% were positive for methamphetamine. (32). A case-crossover research design study noted that cannabis was a trigger for onset of cocaine use even when genetic influences and various environmental conditions were held constant (33).

### Cannabinoids

*Cannabis sativa* contains over 60 cannabinoids as well as over 400 other chemicals including benzopyrene, a known carcinogen. Table 2 lists some of the known cannabinoids (34). The cannabinoids of the *C. sativa* plant include cannabidiol (CBD), cannabigerol (CBG), and cannabinol (CBN) which, in contrast to THC, are not psychoactive. CBD may activate central nervous system (CNS) limbic and paralimbic regions which can reduce autonomic arousal and feelings of anxiety; this is in contrast to THC which can be anxiogenic (35). CBG is found in higher concentrations in hemp and has been used to lower intraocular pressure.

**Table 2:** **Types of cannabinoids**.

Endogenous cannabinoid agonists
2-AG (2-arachidonoyl glycerol)
Anandamide (arachidonoyl ethanolamide)
Cannabidiol (CBD)
Isomer of THC
Cannabinol (CBN)
Metabolite of THC
Cannabigerol (CBG)
Alpha-2-adrenergic receptor agonist
Tetrahydrocannabinolic ACID
THC biosynthetic precursor
Synthetic cannabinoid agonists
WIN 55,212-2
JWH-133
HU-210
CP-55940

Other research also notes that THC and CBD influence different CNS regions and thus, have different effects on cannabis users (36). In addition to anxiolytic effects, CBD has been shown to have anti-emetic, anti-inflammatory, and anti-psychotic effects (37). There is no effect on vital signs (i.e., blood pressure, pulse, body temperature), gastrointestinal transit, or psychological functioning. Doses up to 1,500 mg per day as well as chronic use of CBD have been reported as being well tolerated by humans (37). There can be hepatic drug metabolism inhibition, reduced activities of some drug transporters (i.e., P-glycoprotein), and lowered capacity of fertilization (37).

Euphoria is produced from effects of this lipophilic drug on cannabinoid receptors (ECS: CB 1 and CB 2) in mesocortical and limbic systems; THC also effects the striatum and lateral prefrontal cortex (PFC). Cannabinoid receptors are also found in the liver, gastrointestinal tract, skeletal tissue, and adipose tissue. There are endogenous ligands for cannabinoid receptors (i.e., *N*-palmitoylethanolamide and anandamide) that act like neurotransmitters (27). Part of the complexity of the cannabis issue is the presence of the endogenous endocannabinoid system and this system is now reviewed.

### Endocannabinoid system

The endocannabinoid system consists of CNS cannabinoid receptors and their endogenous ligands; ligands (Latin: binding) refers to triggering molecules that bind to a target protein site (38). Endocannabinoids (endogenous cannabinoid receptor agonists) include arachidonic acid derivatives: 2-AG (2-arachidonoyl glycerol) and anandamide [arachidonoyl ethanolamide (AEA)] (see Table 2) (39, 40). AEA and 2-AG are the two most researched endocannabinoids. The complex effects of this system on emotional and cognitive behavior may be strongly influenced by various environmental factors (41).

The endocannabinoid system is identified both as a cause of psychiatric disorders but also research suggests that proper manipulation of this system may be pharmacologically useful in management of some psychiatric disorders – such as depression, anxiety, anorexia nervosa, and others (42). For example, CBD may be beneficial in treatment of psychiatric disorders (42).

The endocannabinoid system is involved in processes of brain reward that are related to drug abuse – as noted in animal and human research; this includes cue-induced relapse of drug abuse (43). This CNS system is involved in various functions involving memory, emotions, movement, cell proliferation, and other important cell functions (44). Key neuron classes that express high levels of CB1 receptors are GABAergic interneurons in such CNS areas as the cerebral cortex, amygdala, and hippocampus; these areas also contain cholecystokinin, an important neuropeptide (45).

The cannabinoid CB (1) receptor (CB1R) is mainly found in the CNS and the CB (2) receptor is expressed in immune system cells and recent research has shown the importance of this receptor, not only concerning the immune system; both receptors are G-protein coupled receptors and are involved in adenylate cyclase inhibition (46, 47). The CB1R is a G-protein coupled receptor that is associated with most of the CNS endocannabinoid signaling (48). CB1R, also known as CNR1 and CB2R, also known as CNR2 are of importance and future research will most likely show us new information. It is widely found in the cerebellum, basal ganglia, and limbic system; the hippocampus has the highest concentration of cannabinoid receptors. Cannabinoid receptors are found in other tissues including the heart, lungs, endocrine glands, arteries, immune system, sympathetic ganglia, gastrointestinal tract, and reproductive tract (39).

The activation of CB1 receptors can inhibit amino acid and monoamine neurotransmitter release. Certain lipid derivatives [i.e., 2-arachidonoyl glycerol (2-AG) and anandamide (AEA)] can function as endogenous ligands for CB 1 receptors and lead to excitation in such areas as the cerebellum and hippocampus by inhibition suppression (45). Most drugs of abuse alter brain levels of endocannabinoids. Since blockade of this system can change the reward behavior associated with some drugs of abuse, the CNS endocannabinoid system is under active research to develop medications that may be helpful in treatment of drug abuse – including drug relapse (43). Cannabis as potential medication (“medicinal marijuana”) is now considered.

### Use of medical cannabis

Conclusions regarding the “benign” or “malignant” effects of cannabis use influence different countries’ policies regarding legalization or criminalization of cannabis use (49). This is also complicated by use of cannabis products in a “medicinal” manner and thus, effects to find the most “appropriate” variety (varieties) of cannabis that are available (50, 51). A plethora of medicinal benefits have been identified with marijuana use over past centuries (52–54) (see Table 3).

**Table 3:** **Potential benefits of cannabis based on research studies (see text)**.

Remedy for inflammation
Remedy for pain (including chronic pain and neuropathic pain)
Remedy for diarrhea (as in Crohn’s disease)
Treatment for dystonia
Treatment for multiple sclerosis
Treatment for rheumatoid arthritis
Treatment for glaucoma
Treatment for emesis due to chemotherapy
Treatment for epilepsy
Improvement of anorexia in AIDS patients
Treatment for Huntington’s disease
Management of inflammatory bowel disease
Beneficial effect on atherosclerosis
Reduce brain infarct size
Block negative memories in posttraumatic stress disorder
Reduce cardiac reperfusion injury
Adjuvant treatment for prostate carcinoma
Others

The concern over the addictive and psychomimetic qualities of THC placed active research on potential medical benefits of this plant on the backburner until recently (54). However, increased understanding of the cannabinoid signaling system has led to increased research on potential medicinal uses of cannabis (55); this endocannabinoid system, for example, is being analyzed for use in treatment of various neuropsychiatric diseases (44).

Studies are looking at potential benefits of cannabinoids (phytocannabinoids) in management of neuropathic pain, hypertension, post-stroke neuroprotection, multiple sclerosis, epilepsy, cancer, and other disorders (56–63). Cancer research, for example, has identified that cannabinoids can inhibit cancer growth, angiogenesis, and metastasis (63). Cannabinoid-like anti-inflammatory products may be useful as material for wound dressing due to anti-inflammatory effects (64). CBD is under research as a new class of anti-inflammatory bowel disease drug (65).

A number of cannabis products are being manufactured by pharmaceutical companies, including Sativex (THC + CBD), Marinol (dronabinol; THC; Schedule III drug), and Cesamet (THC, Schedule II) (54). The latter two have been approved for use in the anorexia-cachexia syndrome as well as for nausea and vomiting (54). Dronabinol is a synthesized gelatin capsule which has been used to treat glaucoma by lowering intraocular pressure or relieve chemotherapy-induced emesis (*vida infra*) (66). Marijuana has been used to treat the wasting syndrome associated with HIV/AIDS (9). A newer tablet formulation of THC is Namisol (>98% THC) which has been studied to ameliorate pain and spasms in adults with multiple sclerosis as well as relieve nausea, and emesis in HIV or cancer patients (67).

Though cannabis users noted lowering of anxiety and feelings of anxiety, much research remains to be accomplished to identify which if any cannabinoid products may be therapeutically and safely used as pharmacologic management of individuals with anxiety disorders (68). Current research is looking at such anxiety disorders as social anxiety disorder, posttraumatic stress disorders, panic disorder, and obsessive-compulsive disorder (69, 70).

Research suggests that the phytocannabinoid chemical, CBD, may be useful in blocking negative or fear memory associated with posttraumatic stress disorder in a process called *reconsolidation blockage* (71). Some research notes a lower mortality rate among adults with schizophrenia and related psychotic disorders in those who smoked cannabis versus those who did not smoke cannabis (72), while research on smoked, vaporized, and oral cannabis products for potential improvement of health are continuing (73).

### Synthetic cannabinoids (cannabinoid designer drugs; cannabimimetics)

A number of designer drugs have become available in the twenty-first century and cannabis has become involved in this trend as well (see chapter 18). These synthetic cannabinoids (called “legal highs,” “Spice drugs,” “K2” drugs) are similar to THC found in the *C. sativa* plant and produce similar effects to smoking cannabis since they bind to the same cannabinoid brain and peripheral organ receptors as THC (74–80). These herbal blends have been noted since 2008 in various “herbal” smoking products sold via the internet and in retail outlets (called “head shops”) that focus on drug paraphernalia sold for cannabis and other drug use; brand names include such exotic names as *Aroma*, *Yucatan Fire*, *Spice Gold*, and others (74, 81).

Though available in some countries, advertised as “safe,” (since they do not resemble the chemical structure of THC) and considered legal in various locations, they are potentially dangerous drugs – having up to 10 times the strength of delta-9-THC (75, 82, 83). In a moving and dangerous cat and mouse game, sellers change the synthetic cannabinoids in attempts to avoid putting up for sale a specific product identified as illegal in a specific country or area. As one synthetic cannabinoid is banned, others are produced to take their place as there are over 140 different Spice drugs that are produced (74). They may be marked as some type of “incense” or “herbal” product and even “air fresheners” but potential adverse reactions remain (77, 78). Toxicology screens looking at THC may miss the presence of these cannabinoid designer drugs (77, 79).

They are typically tobacco and cannabis free but produce similar cannabis effects that can include withdrawal symptoms, anxiety, intoxication, psychosis, death, and others (*vida infra*) (74, 84). Some reports suggest that increased hallucinations and paranoia are noted with these “spice” synthetic cannabinoids (79). Part of the potential danger is that they can contain various added but often unknown chemicals that are part of the manufacturing process. However, some of the products found on the internet do not have significant amounts of impurities and adverse effects are due to the synthetic cannabinoids themselves and the potential additives (85).

## Cannabis Lab Testing

Those smoking 3.55% pot develop a peak plasma level near 160 mg/ml 10 min after beginning to ingest this product; the THC is removed from the plasma to body tissues leading to its euphoric effects and then to body fat as long-term storage (6). THC is then eliminated over several weeks in the urine and feces. Urine tests can be used to establish the presence of cannabinoid metabolites and can be positive in casual users for up to 10 days versus 14–30 days for chronic pot users (27). Current drug testing (using high-performance liquid chromatography with diode-array detection) can identify low THC content in cannabis seedlings right after germination; however, chemotype determination of THC can occur as the plant ages – at 3 weeks and beyond (86).

Cannabis testing can be used to verify past pot use but not the presence of cannabis intoxication, dependence, or abuse. Testing may also note suppression of testosterone and luteinizing hormone (LH), though it is unclear what such tests actual mean from a clinical viewpoint. Passive inhalation of cannabis does not result in a positive urine test for THC. Urine testing for THC does not identify the presence of synthetic cannabinoids.

Tetrahydrocannabinol-COOH (11-nor-9-carboxy-THC) is the main secondary THC metabolite developed after cannabis is taken; it is not psychoactive but has a long-half life and can be detected for days and in heavy cannabis users, for weeks after consumption. It is an important metabolite used in blood or urine testing for identification of cannabis; urine THC-COOH testing has been used to identify cannabis abstinence and a positive test can be confirmed with gas chromatography-mass spectrometry THC blood testing that indicates recent cannabis exposure (6). Whole blood and plasma testing can also reveal 11-hydroxy-THC after smoking cannabis. Polymerase chain reaction (PCR) testing is available to police to identify where specific *C sativa* samples came from in order to assist with forensic studies and investigations (87). In addition to urine as well as blood testing, saliva and hair testing for cannabis are possible – the latter for evaluation of chronic cannabis exposure (88, 89).

## Medical Adverse Effects

Though some authors question the data on negative effects of cannabis use, most authors conclude that use of cannabis has significant risks for the user in a dose-dependent and/or idiosyncratic fashion (90, 91). Identifying adverse effects of cannabis is challenging because studying an illicit drug can be problematic as can separating out cannabis effects from other drugs that are often taken simultaneously, such as tobacco or alcohol; also, there is variation in techniques of cannabis consumption (90, 92–94).

A case of an infant with altered consciousness after exposure to cannabis smoke (passive inhalation) has been reported (95). Another 10-month-old infant consumed oral cannabis and presented with cannabis poisoning – drowsiness, generalized hypotonia, and restlessness; this infant had high blood as well as urine levels of cannabis products and recovered with symptomatic management that included clinical monitoring for the first 24 h after the ingestion (96).

A number of side effects are possible for the cannabis consumer including increased mortality rates (see Table 4). Chronic use can lead to weight gain from overeating and reduced physical activity. Acute pot use can lead to suppression of rapid eye movement (REM) and diffuse slowing of background EEG activity (27). The smoke of cannabis can be irritating to conjunctival, nasopharyngeal, and bronchial tissue leading to injected conjunctiva, chronic cough, sinusitis, pharyngitis, and (chronic) bronchitis (97). Adolescents or young adults who present with chronic cough should be screened for cannabis use in addition to more classic causes, such as asthma, gastroesophageal reflux, respiratory tract infections, and others (98, 99).

**Table 4:** **Potential adverse effects of marijuana^a^ (see text)**.

Addiction (physiologic)
Withdrawal syndrome
Dependence (psychological) and with heavy use, tolerance
Variety of negative psychological reactions: anxiety, hallucinations, violent behavior, depression, fear
Overt precipitation of psychosis or depression
Insomnia (can be chronic and improved with trazodone)
Memory spans that are impaired
Blunted reflexes
Flu-like reaction (after stopping this drug after 24–60 h, lasting up to 2 weeks)
Confusion and cognition impairment
Alteration of time perception
Amotivational syndrome (lose interest in school or work success)
Physiologic responses can include cough, bronchospasm, bronchitis
Amenorrhea
Immunologic dysfunction

^a^Used with permission from Ref. (14).

Chronic use of cannabis has not been shown to effect thyroid function (100). Acute pancreatitis linked to cannabis use has been reported in a 22-year old male who presented with epigastric pain, nausea, and emesis (101). Abdominal pain due to colonic perforation and subsequent peritonitis has been reported as a complication of cannabis body packing in attempts to illegally smuggle illicit drugs from one country to another (102).

### Cannabis hyperemesis

Cannabinoid hyperemesis is noted in some cannabis users who present with usually sudden, severe, and cyclic (intractable) emesis which resolves with intravenous fluids, antiemetics, and cannabis cessation. There can be cyclic nausea and abdominal pain as well and after a careful evaluation, the cause is linked to cannabis use (103). It was first described in Australia in 2004 and may be missed in patients presenting with hyperemesis and abnormal patterns of bathing (104). Though cannabinoids have been used to treat chronic nausea and emesis, a paradoxical effect on the gastrointestinal tract in noted in cannabis hyperemesis syndrome and three parts are described: prodromal, hyperemetic, and recovery phases (105).

The hyperemesis phase is usually resolved in 48 h. Patients may report temporary symptomatic improvement with prolonged hot showers or bath exposure and thus, compulsive hot water bathing has become part of the cannabis (cannabinoid) hyperemesis complex (103, 106–108). Diagnostic confusion with the cyclic vomiting syndrome may occur (105). If cannabis use resumes, the hyperemesis complex may recur (105, 106, 109).

### Dental effects of cannabis

Pot users tend to have increased risks for dental caries, oral infections, and periodontal disease (110–112). Dysplastic changes and premalignant lesions can be identified in oral mucosa of cannabis users (110). Use of local anesthetics in patients intoxicated with cannabis intensifies and prolongs pot-induced tachycardia (110).

Exposure to smoking (cannabis or tobacco) leads to contact with many carcinogens (pro-carcinogens) such as polycyclic aromatic hydrocarbons (113). Cannabis users often smoke tobacco and drink alcohol which increases carcinogen exposure and risk of oral squamous cell carcinoma which represent 95% of malignant lesions in the mouth (113).

### Pulmonary effects

Some research identifies an anti-inflammatory effect from consumption of the *C. sativa* plant. For example, one study of 5,115 adult males that took place over 20 years noted that occasional and low cumulative cannabis use was not associated with adverse effects on pulmonary function (114). Murine studies suggest that CBD has an immunosuppressive and anti-inflammatory effect on acute lung injury because of an increase in extracellular adenosine (115).

However, it is known that marijuana, as well as tobacco, contains a toxic combination of gases and other substances that can be injurious to the pulmonary system (116). Marijuana smokers usually smoke fewer “joints” than tobacco smokers consume cigarettes; however, methods of cannabis smoking may place more cannabis particulate matter into the lungs than noted with typical cigarette smoking. (116). Those with cannabis dependence will continue to use it despite chronic cough, excessive sedation, or other marijuana-related problems. Combining marijuana with tobacco leads to known tobacco-effects via second-hand smoke.

Pot use can induce some bronchodilation but regular or heavy cannabis consumption can result in generalized airway inflammation with evidence of respiratory epithelial cell injury and damage to alveolar macrophages which can lead to pulmonary infection (116). Sharing of cannabis water pipes has led to the development of pulmonary tuberculosis (TB) (117). Smoking cannabis that contains fungal spores can result in pulmonary aspergillosis in those with immune-compromised conditions (117, 118).

There is a dose-related large airway dysfunction with hyperinflation and obstruction of airflow; one cannabis joint has been noted to be equivalent to 2.5–5 cigarettes in terms of this pulmonary dysfunction (119). Macrophage injury can result in cytokine and nitric oxide impairment. Smokers of cannabis are typically exposed to more carbon monoxide and tar than cigarette smokers; this effect is not related to the THC content (120).

Heavy and/or chronic users of cannabis may have persistent cough, bronchitis (bullous) emphysema [chronic obstructive lung disease (COPD)], pulmonary dysplasia, pneumothorax, TB, and other respiratory infections (27, 116, 117, 121). Cannabis can lead to increased airway resistance and large airway inflammation though causal links to COPD or macroscopic emphysema remain controversial and unproven (93, 94, 119, 122). Smoking both tobacco and marijuana increases risks for abnormal tracheobronchial histopathology and COPD (122).

### Cannabis and cancer

Marijuana smoke contains toxic chemicals in amounts similar to or higher than that found in tobacco and is linked as a potential respiratory tract carcinogen (27, 94, 97, 121). Chronic inflammatory and precancerous airway changes in a dose-dependent relationship as well as increase in airway cancer are reported in cannabis users (91). Anecdotal reports of upper and lower respiratory airway cancer have been published (117, 123). For example, a case of small-cell lung cancer was reported in a 22-year-old male who smoked one marijuana joint three times a week for 3 years (124). However, specific link of cannabis to lung cancer remains unproven (93, 94, 125). Current literature suggests that cannabis-only smokers are at lower risk of lung cancer than tobacco-only smokers (126). However, some epidemiologic data does place an independent role of cannabis smoking in the development of lung cancer (127, 128).

### Cardiovascular effects

Cannabinoids have complex and varying effects on blood pressure depending on which cannabinoid is being studied (34). Acute effects of cannabis include increase in heart rate along with an increase (usually mild) in blood pressure and then decreased vascular resistance-induced orthostatic hypotension (56, 129). Individuals with coronary heart disease may have increased cardiovascular adverse effects from cannabis use (90). Cannabis-induced ST segment elevation mimicking the Brugada syndrome has been reported (130, 131). Other drugs, both licit and illicit (i.e., cocaine), have been linked with the Brugada syndrome as well (132).

Reported cardiovascular effects linked to cannabis include anecdotal cases of acute coronary syndrome, congestive heart failure, and arrhythmias (56, 97, 130, 133–136). Patients with angina may have decreased time with chest pain onset due to the acute effects of cannabis use; also, myocardial infarction may be triggered by the acute effects of cannabis use (56). Patients at high risk for coronary heart disease should be advised to avoid using cannabis (56).

Studies on cannabis also provide evidence of positive or “neutral” effects from cannabis consumption. For example, though inhalation of marijuana may induce acute coronary symptoms, ingestion of cannabinoids may have a positive effect on atherosclerotic heart disease via effects on the endocannabinoid system (135). Also, cannabis use has not been specifically linked to increased hospitalization due to cardiovascular disease or increased mortality from cardiovascular etiology (56). If a patient has a cardiac death and has a positive urine test for cannabinoid, a plasma THC level should also be done before seeking to link the cannabis history with the cardiac death.

### Motor vehicle accidents

Adolescents and young adults who drive vehicles under the influence of pot (often combined with alcohol) are at increased risk (two-times) of motor vehicle accidents leading to potential death and injury (90, 91, 97, 137, 138). Those who consume cannabis without other drugs also place themselves at increased risk for motor vehicle crashes (138). Individuals driving under marijuana influence may experience distortion of on-coming vehicle headlights resulting in motor vehicle crashes. Driving impairment worsens with increasing amounts of cannabis consumed (139). The problem of driving while under cannabis influence is increasing and in some areas of California, for example, the rate of nighttime weekend drivers who tested positive for THC was nearly 20% (140). Unfortunately, tests used in the field to identify if cannabis is involved in motor vehicle accidents may not be sensitive enough to detect the precise presence of this drug (141). Cannabis consumption is also involved in non-traffic injuries, especially falls in the older adult population (142).

### Sports doping

Those involved in sports should understand that cannabis is a drug banned by the World Anti-Doping Agency despite history of pot use in the Olympics and it has been on the list of prohibited drugs of the International Olympic Committee since 1989 (76, 143). Cannabis smoking results in reduced exercise test duration during maximal exercising and increased heart rate at less than maximal exercise levels (56). Cannabis-induced increase in blood pressure and reduced psychomotor activity can also decrease overall athletic performance. Management of urine samples from athletes can be problematic because of the intricacies of interpreting urine samples due to the complexities of prolonged cannabis excretion (144).

## Adverse Effects: Psychiatric

### Cannabis and neurodevelopment effects

Adverse neuropsychological effects of cannabis use must be separated out from the acute effects of cannabinoids, effects of heavy cannabis consumption, and psychiatric disorders worsened or even caused by cannabinoids (145). Specific effects in individual cannabis users are difficult to predict because of the heterogeneity or non-uniformity of different studies that have been done to seek neuroimaging effects in cannabis use; some authors have concluded it is difficult to prove major effects of cannabis on brain structure (146, 147). Some research suggests that identified cannabis-related neurocognitive performance defects disappear after 25 days of cannabis abstinence (148).

However, other recent research studies are noting that cannabis users demonstrate important deficits in prospective memory and executive functioning that exist beyond acute cannabis intoxication (149). The presence of cannabis and nicotine use disorders in parents appears to increase the risk for major depressive disorder in their late adolescent offspring (150). Studies in animals and humans suggest a subtle (versus gross) effect on cognitive functioning with later development of hyperactivity, reduced attention span, impulsivity, depression, and substance use disorders (151). Most frequently reported adverse effects of cannabis use include mental slowness, reduced reaction times, and increased anxiety (90). Dysfunction occurs to dopamine and opioid neurotransmitter systems (152).

Animal and human research concludes that the developing brain, with its high neuronal plasticity, is vulnerable to exposure to exogenous cannabinoids, particularly in the perinatal/prenatal period and during young adolescence (44, 152–155). Animal and human studies suggest that *early* onset of cannabis use (i.e., early adolescence) can increase risks for cognition dysfunction, CNS changes (i.e., low striatal dopamine release), neuropsychiatric disorders, cannabis dependence, and consumption of additional illicit drugs (153, 156). Persistent or problematic marijuana use can lead to major interference with daily life activities whether at work, in school, or in one’s home.

Cannabis use often develops in adolescence and early adulthood which, as noted, is a vulnerable time for subsequent adverse brain effects (48). For example, cannabinoid receptors are abundant in the CNS white matter in adolescence as well as young adulthood; long-term cannabis use during this time period can lead to impaired axonal fiber connectivity with negative effects on the white matter of the brain (157). Cannabis use in early adolescence may alter CB1R signaling with potentially increased risk for development of psychiatric disorders (48). Early adolescent cannabis use has been linked with adolescent drop-out behavior in some research (158).

Some research links other CNS problems with heavy cannabis use. For example, one study identified regional brain abnormalities (in the hippocampus and amygdale) in long-term, heavy pot users – data found in both human and animal studies (159). Animal studies noted hippocampal-dependent short-term memory deficits that occurred in some but not all rats given chronic cannabinoid administration (160).

### Cannabis and ADHD

One report of 162 adolescents studied during inpatient management of problems related to drug dependence (i.e., marijuana, heroin, alcohol, or cocaine abuse) revealed attention deficit hyperactivity disorder (ADHD) in 34% of them (161). A study from different medical centers that focused on 600 adolescents (ages 13–16 years of age) who were undergoing management for marijuana-related problems reported that 38% also had ADHD (162). Such studies support the theory that ADHD and adolescent substance abuse disorder (including cannabis dependence) can be seen as a developmental disorder with similar underlying physiologic mechanisms (20).

### Cannabis dependence

Psychological dependency and tolerance are classically described in pot smokers. The American Psychiatric Association’s DSM-IV-TR describes two cannabis use disorders (cannabis dependence and abuse) and six categories of cannabis-induced disorders (see Table 5) (27), while DSM-V has included cannabis withdrawal. Some have placed the dependence rate at 7–10% of regular users (91), while a susceptibility gene, NRG1, has been associated with cannabis dependence in African Americans (163).

**Table 5:** **DSM-IV-TR cannabis use and induced disorders (27)**.

Cannabis use disorders
Cannabis dependence
Cannabis abuse
Cannabis-induced disorders
Cannabis intoxication
Cannabis intoxication delirium
Cannabis-induced psychotic disorder, with delusions
Cannabis-induced psychotic disorder, with hallucinations
Cannabis-induced anxiety disorder
Cannabis-related disorder not otherwise specified

Those with cannabis dependence can consume cannabis in potent forms for years and spend several hours a day in finding as well as taking it; there can be physiologic dependence and also psychologic dependence (27). Cannabis intoxication may last longer with oral cannabis versus smoking it. Intoxication may even last up to 24 h due to effects of enterohepatic circulation and/or slow release of fat soluble THC and other cannabinoids from fatty tissue. Cannabis use disorders are more common in males versus females and are most prevalent in the 18- to 30-year-old group (27).

Depersonalization and derealization episodes are described in pot users (27). Anecdotal cases of cannabis-induced depersonalization in adolescents have been reported (164). A history of conduct disorder in childhood or adolescence and antisocial personality disorder are risk factors for substance use disorder including cannabis-related disorders (27). Differentiation of cannabis-induced disorders from primary mental health disorders can be difficult. Ataxia and aggression are more likely to be seen with phencyclidine (PCP) intoxication versus cannabis intoxication; aggression with nystagmus or ataxia is more likely to be from alcohol intoxication (27).

Demonstrating a direct link between cannabis use and the development of overt depression has been problematic and not clearly proven (165). Regular cannabis consumption (particularly daily use) in adolescence has been linked with increased risks for anxiety disorders in adolescents and young adults, even after the cannabis was stopped (155). Anxiety is linked to regular or heavy use of cannabis and further research is needed to unravel this connection in more detail (166). For example anxiety can arise due to fear of being discovered by law enforcement officials; there can be episodes resembling panic attacks.

### Cannabis withdrawal syndrome

Chronic pot users can develop psychological addiction and a withdrawal syndrome comparable to heroin addiction (8). A heavy marijuana user, whether an adolescent or adult, who suddenly stops this drug can develop a recognizable withdrawal syndrome as reported by various research studies (167–173). Withdrawal symptoms can develop within 48 h of cessation and include irritability, restlessness, anxiety, aggression, and sleep difficulties. (90). Withdrawal symptoms tend to subside in 2–12 weeks after cannabis abstention (90). As noted, a CNR1 gene has been linked with abstinence-induced withdrawal symptoms (174). Heavy cannabis use has been linked with a smaller amygdala and hippocampus while those with CNR1 may be predisposed to smaller hippocampal volume following heavy cannabis use (174). Some smoke pot to control problems with anger (170–173).

Withdrawal symptoms are due to the cessation of THC and are relieved with taking delta-9-THC or simply smoking marijuana (167, 175). Adolescents undergoing treatment for cannabis dependence experience withdrawal symptoms most acutely during the first week of cannabis absence and this tends to ease over the next month of abstinence (167, 176). Research notes that the majority of heavy pot users report several symptoms after stopping cannabis and this can complicate effective management, especially since cannabis-dependent users may resort to consuming various others drugs to relieve the symptoms of cannabis withdrawal (167, 177). Diagnostic criteria for a formal cannabis withdrawal syndrome (CWS) have been included in the fifth edition of the American Psychiatric Association’s Diagnostic and Statistical Manual of Mental Disorders (DSM-V) (178).

### Addiction

The identification and isolation of THC in 1964 enhanced specific research into effects of cannabis on humans and animals (44). Endogenous cannabinoids (particularly CB1 receptor activation) activate neural mechanisms in the CNS similar to how other reward-enhancing drugs induce drug addiction (179–181). This core reward system is part of the addiction mechanism that use of cannabis develops in the unwary pot smoker as well as in users of other illicit drugs of addiction (182, 183). This mechanism involves the meso-accumbens reward circuitry of the CNS as well as neural firing of the neurotransmitter, dopamine. Some research notes that THC can induce striatal effects on dopamine as noted with other drugs of abuse (184). Though some studies suggest dependence develops faster in cocaine versus cannabis users, recent research concludes there is no such difference (185).

Addiction also involves PFC dysfunction with frontostriatal dysfunction and the erosion of the ability to stop the addiction; more research is needed to identify potential impact of cannabinoids in the PFC and its potential role in cannabis dependence (186–188). A nucleotide polymorphism has been identified in a cannabis receptor-1 gene (CNR1) – rs2023239 – that is associated with cannabis dependence, cannabis craving, and withdrawal symptoms due to cannabis abstinence (174). Some neuropeptides called orexins (hypocretins) originate in the lateral hypothalamus and are linked to features of drug addiction (i.e., cannabis, nicotine) that include drug craving, relapse, and withdrawal (189).

### Cannabis and psychosis

Chronic use of cannabis, particularly with the newer synthetic cannabis products, is associated with increased rates of psychosis (190–192). Frequent cannabis use increases the risk by two times for schizophrenia and psychotic symptoms, perhaps by cannabis-induced disruption of the endocannabinoid system in which the normal signaling and functioning of this endogenous system is disturbed (193). Cannabis (marijuana) is commonly used by those with schizophrenia and can cause paranoia in approximately 40% of persons experimenting with this drug (194). Patients with schizophrenia who consume cannabis are hospitalized at rates higher than those who do not use cannabis (195).

Cannabis-induced schizophrenia is currently theorized as being due to dysfunction of late postnatal brain maturation in which glutamatergic transmission dysfunction leads to abnormal prefrontal neurocircuitry (196). Exposure of adolescents to cannabis at certain times in adolescence and at certain doses may lead to prefrontal cortical circuitry abnormalities with the result of inducing schizophrenia in susceptible adolescents (196).

One research group reports a mean time of 7.0 ± 4.3 years between onset of cannabis use and onset of psychosis (191). Those at risk for psychosis may be vulnerable to brain volume loss (i.e., cerebellum, PFC, and cingulate) due to use of cannabis (197). Self-mutilation can also occur with cannabis-induced psychosis (198). Those with psychosis who use cannabis may not notice improved psychotic symptoms with cessation of their cannabis (199).

Most cannabis users do not develop psychosis and this cannabis-psychosis link appears to be via a complex environmental-genetic-molecular interaction possibly involving anandamide dysfunction and other biological factors (192, 200, 201). Most research suggests a link between cannabis use and risk for suicide in patients with psychosis and also those without psychosis (202). The development of schizophrenia and use of cannabis share a variety of similarities, including neuropsychological deficits, reduced motivation, hallucinations, and initiation in late adolescence (203).

Cannabis is also a very common illicit drug for individuals with psychosis and disruptive disorders to consume (13, 204). Those with psychosis have a higher rate of cannabis use than the general population, probably in attempts to utilize the cannabis-induced euphoria to deal with negative aspects of schizophrenia, such as boredom and depression (12). Cannabis can also prompt the onset of psychotic symptoms in otherwise healthy people as well, and this includes paranoia and/or delusional thinking due to effects of THC on striatal and prefrontal function.

Thus, use of marijuana can have opposite effects on different users. Some research fails to find a clear association between use of cannabis and symptoms of psychosis, especially with low or moderate cannabis use (205). A major component of cannabis, CBD, has been shown by some research to have *anti*-*psychotic* effects (206). The presence of CBD may explain the lack of psychosis development in many cannabis users. Studies with CBD (versus THC) suggest a modulating effect based on functional MRI brain imaging (207). Schizophrenic patients using cannabis may be particularly sensitive to brain damage from the cannabis though CBD may provide a protective effect from brain volume loss (203).

## Management

### Behavioral therapies

The “hedonic” CNS dysregulation seen in drug addiction studies in animals and human subjects underscores the concept of drug addiction as a brain disease and the necessity of *prevention* before this complex brain dysfunction arises (12, 183, 208). Indeed, it is difficult to convince an addicted cannabis user to stop smoking this plant and thus, *prevention* via intensive and comprehensive education is the best option currently available. Unfortunately cannabis dependence is difficult to treat successfully and few who seek to stop their cannabis addiction succeed in long-term cessation (181). One of the ironic reasons for this is that chronic cannabis consumers classically have cognitive impairments which lead to defects in their decision making skills (209).

However, there are steps therapists can take in helping this phenomenon of a nation and a world obsessed with smoking pot. Continued education of the dangers of cannabis should take place on a persistent and relentless basis. It should be understood that some literature and some people conclude that the negative impact of smoking cannabis is limited (210). Cannabis consumers may be cognizant of such literature, which may encourage them to continue to use this so-called safe illicit drug. Thus, teaching about the potential problems of cannabis should always be provided by healthcare providers to help offset this destructive message of “benign” pot consumption.

Treatment specifically for cannabis use is needed as management of other or co-existing drug dependence (such as heroin, tobacco, or alcohol, for example) does not necessarily reduce levels of cannabis consumption (211). Comprehensive school-based prevention programs teaching about drug use, including cannabis, can be beneficial in lowering youth drug experimentation and addiction (212, 213). Increased education about high-risk adolescent behaviors for adolescents and their parents can be useful in prevention efforts (214). Intense education is needed for groups at high risk for drug abuse, such as males, young adults, and individuals with increased psychological stress (215).

Behavioral principles for management in adolescence can be directed by factors shown to suggest higher risk for cannabis use including genetic factors, family history, minimal parental supervision, drug availability, high-risk peer group, and those with a need for higher thrill-seeking activities (216, 217). Groups at high risk of mental health problems are those with combined drug use, such as comorbid cannabis and methamphetamine; thus, this group should receive intense management (218). Comprehensive behavioral management can also be of help in reducing cannabis use in those arrested for cannabis possession (219). Screening of these persons for suicide risk is recommended and lower cannabis use can reduce risks for suicide in those with and without psychosis (202). Patients with psychosis and cannabis smoking should undergo behavioral therapies to improve both problems and not just one (220).

If life-time abstinence from cannabis is not possible, delaying its use (especially heavy use) as long as possible after adolescence and young adulthood, may result in less white matter damage from cannabis consumption than noted during cannabis smoking in the second and third decade of life (157). Management can also focus on reducing cannabis use if abstinence is not possible, since adverse effects tend to be increased with heavy use of cannabis (92). Therapy should seek underlying factors in use of cannabis or other drugs. For example, research notes that socially anxious males are at increased risk for use of cannabis as part of a mechanism to deal with or avoid social situations (221). Thus, therapy can be directed at reasons for this avoidance behavior and the acquisition of successful strategies to improve their social anxiety which may reduce the need for ongoing cannabis consumption.

Often recommended behavioral management strategies (such as cognitive-behavioral therapy and contingency management) have their limitations and combination of these techniques does not increase success in helping those with cannabis dependence (222). However, research notes that youth with cannabis use disorder benefit more from CBT (versus family therapy) if they are older teenagers (i.e., 17–18 years of age) and do not have pre-existing psychiatric disorders; in the same study, multidimensional family therapy was more helpful (versus CBT) for those who were younger (i.e., under age 17) and/or had a past year history of disruptive disorders (i.e., opposition defiant or conduct disorder) (223).

However, one should continue to provide the cannabis consumer with hope and encouragement that overcoming this drug addiction is possible despite its wide acceptance in society. Research does show that such benefit is more likely with intensive or prolonged behavioral therapy, especially cognitive-behavioral therapy and motivational interviewing (224). Also, it should be understood that even brief (i.e., four) sessions of motivational interviewing with cognitive-behavioral therapy delivered via telephone can be beneficial, at least in the short run, for *motivated* cannabis users who called in seeking help with treatment (225).

Behavioral therapies can be useful in the motivated cannabis addict and such counseling seeks to help the cannabis consumer gain control over their addiction at the CNS level by enhancing neuroanatomical progression from ventral striatal (nucleus accumbens) to dorsal striatal control (183). Even brief interventions may have positive benefit in less cannabis use at 3 month follow-up (3) and another study at 12-month follow-up (17).

Also, therapy seeks to help the addict deal with cannabis craving and relapse triggers, such as becoming re-exposed to various drugs of addiction; it is also important to help this person deal with stress and also with finding and avoiding old clues in his/her milieu, such as key persons, places, or objects (183, 208). Genetic factors also have a role in why some develop addiction and are recalcitrant to management strategies (208). Involvement of families, schools, communities, and peer groups are critical in seeking to reduce and prevent cannabis smoking in adolescents. It is unclear what the interactions are between abuse of illicit drugs and use of prescription drugs, but the potential drug interactions should be considered in prevention and treatment plans (226).

## Pharmacologic Therapies

There are currently no FDA-approved pharmacologic agents for management of cannabis dependence. Traditionally, pharmacologic agents have not been specifically beneficial in treating marijuana addiction whether dependence or withdrawal symptoms (227). However, a careful assessment of each patient is necessary and a reduction in cannabis use can be noted in some patients under standard pharmacotherapy for mental illness (224). There is no evidence that one anti-depressant, anxiolytic, or anti-psychotic is more effective than another. Cannabis use disorders are common in those with schizophrenic spectrum disorders; however, there is no current literature that guides clinicians in the best treatment approaches for this dual diagnosis (228). Knowledge, however, is slowly emerging to guide pharmacologic therapies for cannabis-induced problems in the twenty-first century.

Individuals with co-occurrence of cannabis and tobacco use tend to have higher abstinence rates if treatment includes measures aimed at dual abstinence (25). Thus, pharmacotherapy for nicotine addiction may help the individual stop marijuana use as well by removing use of and thus, influence of tobacco. Pot-associated persistent insomnia may be improved with use of trazodone. Research is looking at new methods of treatment that will emerge from the study of the genetics of addiction including gene-milieu interplay and role of genetic variation (229).

## Cannabis Intoxication

Pharmacologic management typically centers on use of benzodiazepines or atypical anti-psychotics. Propranolol and rimonabant (*vida infra*) have been reported to be beneficial in management of acute, physiologic effects of cannabis *intoxication*; use of flumazenil and CBD is under current study in this regard (230).

## Cannabis Withdrawal

Oral THC (dronabinol) may relieve cannabis *withdrawal* symptoms but not relapse (231, 232). Adding dronabinol and lofexidine (alpha-adrenergic receptor agonist) can lower the severity of withdrawal and lower relapse rates in those with cannabis dependence (233). The anti-depressant mirtazapine can also help those undergoing cannabis withdrawal.

## Cannabis-Associated Psychosis

Cannabidiol has been shown in animal and human studies to have anti-psychotic effects and it may become a useful pharmacotherapeutic agent for schizophrenia management (206). Research has therapeutically targeted the cannabinoid (CB1) receptor since delta-9-THC is a partial CB1 receptor agonist. Treatment of patients with psychotic disorders and cannabis use should include seeking to reduce or stop the cannabis use, perhaps using cannabinoid agonist medication (220). Those with psychosis can be provided with appropriate anti-psychotic medications.

## Cannabis Dependence

Nabilone is a synthetic THC analog with improved bioavailability over dronabinol and is under current research as a potential medication for marijuana *dependence* as it may lead to a positive mood and limited adverse cognitive effects in marijuana smokers (234) (see chapter 5). Olanzapine can reduce psychomimetic effects of THC in some individuals and its potential benefit is under study (235). Rimonabant and the anxiolytic buspirone have provided some efficacy in cannabis addicts on maintenance therapy. Rimonabant is a CB1-selective cannabinoid receptor antagonist/inverse agonist that has been under research for obesity treatment as well as treatment of nicotine and marijuana addiction. It is not available in the United States and was pulled from the European market in October of 2008 due to a high risk to benefit ratio; concerns have been identified regarding major psychiatric adverse effects (i.e., depression and suicide) as well as overall efficacy (167).

### Oxytocin

Animal research notes that various drugs, including cannabis, can induce chronic changes in markers of oxytocin function with resultant social behavior dysfunction. Oxytocin is a neurohypophyseal hormone that has been under research in the regulation of drug abuse (236). This neuropeptide may serve as a neuromodulator on neurotransmission of dopamine in the nucleus accumbens as well as effects on the hippocampus (236). CNS oxytocin pathways may offer a way of improving mood and social deficits found in some individuals with drug addiction (237). Research suggests that use of intranasal oxytocin may be useful in correcting the negative cannabis (and other illicit drug) effect on social behavior and perhaps protect the individual from addictive disorders. (238).

### *N*-acetylcysteine

*N*-acetylcysteine (NAC) has been shown to be useful in management of cannabis-dependent adolescents and young adults due to modulation of glutamate in the nucleus accumbens; it is suggested as an adjuvant treatment (1,200 mg per day) along with psychological management (239).

### Others

Research has failed to validate the use of *naltrexone* for the treatment of cannabis dependence. *Entacapone* is a member of the drug class called nitrocatechols that is used in the management of Parkinson’s disease. It is an inhibitor of COMT (catechol-*O*-methyltransferase) and is under research as a possible drug for cannabis dependence.

Other drugs under research include lithium, dronabinol, URB597 [fatty acid amide hydrolase (FAAH) inhibitor], methyllycaconitine, or MLA (nicotinic alpha-7-receptor antagonist), and endocannabinoid metabolizing enzymes (240).

## Summary

Research must provide more specific information on how cannabis and products derived from cannabis can be useful in medical management of illness or if the cannabis risks simply outweigh any potential benefit (241). Does cannabis have advantages over specific cannabinoids? Can the psychotropic effects of cannabis be reduced, while utilizing potential medicinal benefits of CB1 receptors activation? Oral cannabis undergoes changes which produce a narrower therapeutic window and raises questions regarding whether or not medicinal cannabis products can be given orally or must be used as a mist or smoke for positive and optimal benefit. It is difficult to find an oral dose that benefits most without induced unacceptable or unwanted adverse effects in many unwary consumers (241).

Generalized consumption of the unprocessed *C. sativa* plant can lead to considerable public health risks including increased schizophrenia, psychosis, dependence, and other risks as noted in this review (242). A medical role for specific cannabinoid compounds remains under active medical research (243, 244). What is well-known is that there are many potential medical and psychiatric adverse effects to smoking cannabis and its synthetic derivatives. Links between use of alcohol, cannabis, and other illicit drugs continues to be unveiled by research (245). Research is identifying new techniques of cannabis identification to assist the police and other authorities in forensic investigation (246). Finally, treatment of an individual with cannabis dependence is very difficult and requires more research in the twenty-first century (247, 248). Indeed, *C. sativa*, a controversial plant known for thousands of years, remains disputatious and contentious in the twenty-first century (249).

## Conflict of Interest Statement

The authors declare that the research was conducted in the absence of any commercial or financial relationships that could be construed as a potential conflict of interest.
